# Associations of social network use and social network addictive behaviors with self-esteem in adolescents: the EHDLA study

**DOI:** 10.3389/fpsyt.2025.1499679

**Published:** 2025-03-26

**Authors:** Estefanía Martínez-Iniesta, José Adrián Montenegro-Espinosa, Bruno Bizzozero-Peroni, Jorge Olivares-Arancibia, Rodrigo Yáñez-Sepúlveda, Daniel Duclos-Bastías, Josefa María Panisello Royo, Arthur Eumann Mesas, José Francisco López-Gil, Estela Jiménez-López

**Affiliations:** ^1^ Health and Social Research Center, Universidad de Castilla-La Mancha, Cuenca, Spain; ^2^ One Health Research Group, Universidad de Las Américas, Quito, Ecuador; ^3^ Instituto Superior de Educación Física, Universidad de la República, Rivera, Uruguay; ^4^ AFySE Group, Research in Physical Activity and School Health, School of Physical Education, Faculty of Education, Universidad de Las Américas, Santiago, Chile; ^5^ Faculty Education and Social Sciences, Universidad Andres Bello, Viña del Mar, Chile; ^6^ iGEO Group, School of Physical Education, Pontificia Universidad Católica de Valparaíso, Valparaíso, Chile; ^7^ IGOID Research Group, Faculty of Sport Science, University of Castilla-La Mancha, Toledo, Spain; ^8^ DigimEvo, Barcelona, Spain; ^9^ Centro de Investigación Biomédica en Red de Salud Mental, Instituto de Salud Carlos III, Madrid, Spain

**Keywords:** psychological well-being, adolescent behavior, self-concept, social media, addiction, youth

## Abstract

**Objective:**

The aim of the present study was to examine the relationships of social network (SN) use and SN addictive behaviors with self-esteem in Spanish adolescents. The use of specific social media platforms and their associated addictive behaviors related to self-esteem were also evaluated.

**Methods:**

This cross-sectional study used secondary data from the Eating Healthy and Daily Life Activities (EHDLA) project, which included a representative sample of adolescents aged 12–17 years from the *Valle de Ricote* in the Region of Murcia, Spain. The sample consisted of 632 adolescents. Addiction and SN use were assessed via the Short Social Media Addiction Scale, and self-esteem was assessed via the Rosenberg Self-Esteem Scale. Generalized linear regression analyses with Gaussian distributions were conducted to calculate unstandardized beta coefficients (*B*) and their 95% confidence intervals (CIs). Sociodemographic, lifestyle, and anthropometric data were included as covariates.

**Results:**

In terms of SN use, Instagram was the most accepted, in contrast to Facebook, which was the least used. The most notable addictive behaviors toward SN use included salience (i.e., when using SNs is the major concern and the priority motivation) and tolerance (i.e., when increasing use is desired). Furthermore, self-esteem levels decreased as adolescent’s addiction levels increased. In the adjusted model, an increase in each addictive behavior was associated with a significant decrease in self-esteem (*B =* -0.699; 95% CI -0.890 to -0.508; *p <* 0.001). Individually, there was a negative and significant association between Twitter use and self-esteem (*B* = -0.356; 95% CI -0.695 to -0.017; *p* = 0.040). With respect to addictive behaviors toward SNs, mood modification showed the strongest negative association with self-esteem (B = -2.580; 95% CI -3.192 to -1.968; *p* < 0.001), followed by conflict (B = -1.410; 95% CI -2.147 to -0.673; *p* < 0.001), relapse (B = -1.350; 95% CI -1.999 to -0.701; *p* < 0.001), tolerance (B = -0.928; 95% CI -1.596 to -0.260; *p* = 0.007), salience (B = -0.892; 95% CI -1.623 to -0.161; *p* = 0.017), and finally, withdrawal behaviors (B = -0.170; 95% CI -1.905 to -0.435; *p* = 0.002), all of which were significantly negatively associated with self-esteem in adolescents.

**Conclusions:**

SN use and SN addictive behaviors could significantly affect adolescent’s self-esteem. The findings suggest that the use of Twitter and certain addictive behaviors, such as tolerance, salience, mood modification, relapse, withdrawal, or conflict are particularly linked to lower self-esteem. These results highlight the need to develop strategies aimed at promoting healthy engagement with SN platforms and fostering adolescent’s psychological well-being.

## 1 Introduction

In recent years, self-esteem has gained importance in research as a factor that could be critical for psychopathologies such as anxiety and depression (1). Self-esteem has been defined as the subjective evaluation that individuals hold toward themselves (2). Self-esteem has been shown to be a fundamental component of emotional and psychological development during childhood and adolescence (2), playing a critical role in overall well-being and coping abilities (3). Thus, its enhancement has been considered an important target for interventions aimed at improving physical and mental health (1).

While children and adults also engage with social network (SN) use, adolescents seem particularly vulnerable to their influence due to several factors (4). Adolescents have greater access to SN platforms, with 98.5% of Spanish adolescents registered on at least one platform and 83.5% using more than three platforms (5). In this sense, SN use has become an inescapable part of the lives of adolescents today (6). Digital platforms, such as Instagram and TikTok, offer opportunities for social connection, self-expression, and access to vast amounts of information (7). However, these platforms also raise concerns about their impact on adolescent’s self-esteem, particularly in relation to idealized images of body image and peer comparisons (8). Adolescence is a stage of life marked by intense physical, emotional, and social changes. The adolescent brain is still developing, and research suggests that, compared with children or adults, adolescents are more susceptible to the influences of SNs (9). Similarly, this period is characterized by heightened concerns about body image and the shaping of self-perception on the basis of external feedback (2). This suggests that adolescents can be particularly sensitive to acceptance and rejection of SNs, as their heightened emotional sensitivity and the ongoing development of their reflexive thinking and cognitive control make them especially responsive to emotion-provoking content (10). Therefore, increased access, combined with the intense desire for social validation and the formation of self-identity, makes adolescence a particularly sensitive developmental stage for studying self-esteem and the potential impact of an especially prevalent factor in this period, such as the use of SNs (11).

Given the complex relationship between SN use and self-esteem, it is important to account for other key factors, such as sex, age, socioeconomic status, and lifestyle habits, which may be related to the impact of SN use on adolescent’s self-perception and well-being (12–15). Among the factors that can influence self-esteem, body perception can be especially important, often leading to high levels of concern about appearance and body image (16). While SNs can offer numerous benefits, their misuse could contribute to various problems that impact well-being (11), as well as physical, cognitive, and emotional health issues (17). In the online world, much communication occurs through images, which serve as a form of virtual introduction (18). The idealization of the physical appearance of SNs can lead to the promotion of unrealistic beauty standards, especially concerning body image. Adolescents are exposed to a continuous stream of idealized images of the “perfect” body, which can negatively affect their self-esteem, as they compare themselves to these unrealistic standards (19). At the same time, SN use carries certain risks, such as bullying, loss of privacy, and the potential emergence of behaviors and symptoms resembling those observed in drug addiction, such as tolerance, withdrawal symptoms, relapse, mood modification, salience, or conflict with other areas of life (20). In this sense, although SN addiction is not recognized as an addictive disorder in the current Diagnostic and Statistical Manual of Mental Disorders (21), there is considerable evidence supporting the existence of addiction behaviors regarding the use of SNs (22), favored by the particularly appealing nature of these platforms, which are easily accessible and act as immediate reinforcers—key factors in addictions (23). These behaviors have been linked to negative outcomes in self-esteem and psychological well-being, as adolescents modify their behaviors to meet the pressure of using these platforms (24).

Research has shown that negative peer evaluations, such as criticism of body image, clothing, or social activities, increase the risk of body dissatisfaction and other related disorders (25). Moreover, addictive behaviors toward SN use have become an emerging area of concern (26, 27) in which mixed results have been reported, with some studies indicating that the use of SNs may increase self-esteem in adolescents (28, 29). Therefore, The aim of the present study was to examine the relationships of SN use and SN addictiive behaviors with self-esteem in Spanish adolescents, addressing the existing gap in the literature by examining how these behaviors interact in the adolescent population. We hypothesized that increased SN use is associated with lower self-esteem in adolescents and that addictive behaviors related to SN use are negatively associated with self-esteem in adolescents.

## 2 Materials and methods

A cross-sectional study was carried out using secondary data from the Eating Healthy and Daily Life Activities (EHDLA) study (30). A total of 632 adolescents (56.0% girls) were included in this study. The original EHDLA study included a representative sample of adolescents aged 12–17 years from the three secondary schools in *Valle de Ricote* (Murcia region, Spain) from 2021–2022. However, since this study uses secondary data from the EHDLA study, the representativeness of the sample is not necessarily guaranteed in the current analysis. The detailed methodology of the EHDLA study has been published previously (30). To participate in this study, parents or legal guardians of the adolescents received a written informed consent form, which they signed before enrolling their children. In addition, the adolescents were asked if they were willing to participate in the study after being provided with an information sheet explaining the purpose of the research project, as well as the tests and questionnaires used.

The participants had to meet the following conditions to participate in the study: 1) be between the ages of 12 and 17 years and 2) live or attend secondary schools in *Valle de Ricote*. Certain exclusion criteria were also established, including 1) exemption from physical education classes, since the tests and questionnaires were carried out during the development of the same class; 2) presenting any health condition that restricted physical activity or required special attention; 3) being under pharmacological treatment; or 4) lacking authorization from parents or legal guardians. This research project received approval from the Bioethics Committee of the *Universidad de Murcia* (2218/2018) and from the Ethics Committee of the Hospital Complex and Integrated Healthcare Management of *Universidad de Albacete* (2021–85). Similarly, this study adhered to the Declaration of Helsinki and guaranteed the protection of the human rights of the participants who were included.

### 2.1 Variables

#### 2.1.1 SN use (independent variable)

The adolescents completed the interview on a scale in which SN use (i.e., Facebook, Twitter, Instagram, Snapchat, and TikTok) was evaluated. The scale consists of measuring the frequency of use with five possible responses: (a) “I never or rarely use them”; (b) “I am a low consumer”; (c) “I am a medium consumer”; (d) “I am a fairly high consumer”; and (e) “I am a very high consumer” (31). For this study, the responses were categorized into numerical values from 0 (“never or rarely use”) to 4 (“I am a high consumer”). SN items were summed to obtain a total score (ranging from 0–25, Cronbach’s alpha [*α*] = 0.60), where higher scores indicated greater use (32). WhatsApp was evaluated with the same scale but was not included in the total score because it is a messaging application.

#### 2.1.2 Addictive behaviors toward SNs (independent variable)

The Short Social Networks Addiction Scale-6 Symptoms (SNAddS-6S) was used to assess possible addiction to SN use (33). The SNAddS-6S was developed from a review of the literature on SN addiction and psychological theories of addiction. The SNAddS-6S is composed of 18 items and five different factors (time-management, mood modification, relapse, withdrawal, and conflict), with the time-management factor as a higher-order factor integrated by salience and tolerance as subfactors. The Short SNAddS-6S is composed of six items and has a unifactorial structure. The six dimensions of the SNAddS-6S are a) tolerance (measures how individuals need more time or interaction to experience the same level of gratification); b) salience (assess the increasing importance of SN in an individual’s daily life to the point where they dominate their thoughts; c) mood modification (reflects how individuals use SNs to modify their emotional state [e.g., to relieve stress or enhance positive emotions]); d) relapse (describes the tendency to return to addictive behavior despite previous attempts to reduce or stop SN use); e) withdrawal (indicates negative emotions (such as anxiety or irritability) experienced when SN use is limited or unavailable); and f) conflict (measures the negative impact of SN use on other areas of life, such as relationships, work, or academic performance). Additionally, the SNAddS-6S demonstrated good internal consistency, with a Cronbach’s α of 0.72, which is considered acceptable for social science instruments. This finding supports the scale’s reliability and indicates that the items measure a consistent construct of SN addiction.

#### 2.1.3 Self-esteem (dependent variable)

Self-esteem was measured via the Rosenberg Self-Esteem Scale (2), which consists of 10 items on a 4-point Likert scale, where 1 means “not true for me” and 4 means “very true for me”. The total score varies between 10 and 40 points, and the ranges are low self-esteem: 10–24; medium self-esteem: 25–29; and high self-esteem: 30–40 (34). The Spanish version of this scale was used, which demonstrated reliability in its initial validation in relation to adolescents (2) and, in addition, high levels of internal consistency and temporal stability (35).

### 2.2 Covariates

#### 2.2.1 Sociodemographic data

Each participant stated their sex and age. The socioeconomic status of the adolescents was assessed via the Family Affluence Scale (FAS-III) (36), which comprises six questions with answers ranging from 0 to 13 points, summed to obtain a total score, where higher scores represent higher socioeconomic status.

#### 2.2.2 Lifestyle variables

To assess the participant’s sleep duration, they were asked about their usual bedtime and wake-up times on both weekdays and weekends. The mean daily sleep duration was calculated via the following formula: [(mean duration of nighttime sleep on weekdays ×5) + (mean duration of nighttime sleep on weekends ×2)]/7. To collect information on physical activity and sedentary behavior among adolescents, we used the Spanish version of the Youth Activity Profile (YAP-S), which was previously validated and adapted for use in Spanish adolescents (37). This self-administered questionnaire consists of 15 items designed for young people aged 8–17 years. This questionnaire uses a 5-point Likert scale and is divided into three sections: school activity, extracurricular activity, and sedentary habits. School activity includes physical education classes, lunchtime, recess, and transportation. Extracurricular activities include activities before and after school, evening, and weekend activities. Sedentary habits assess the time spent watching TV, playing video games, using a computer or cell phone, and overall sedentary time. Physical activity and sedentary behavior scores were calculated by summing the corresponding items in each section (37).

The Mediterranean Diet Quality Index in Children and Adolescents (KIDMED) questionnaire was used to measure adherence to the Mediterranean diet (38). This assessment tool consists of 16 questions that explore various parameters of the Mediterranean diet, such as the consumption of fruits, vegetables, legumes, fish, dairy products, and cereals and eating habits such as breakfast and fast-food consumption. Each affirmative response related to adherence to the Mediterranean diet is scored +1; conversely, negative responses related to adherence to the Mediterranean diet are scored -1. The sum of these scores classifies participants into three categories of adherence to the Mediterranean diet: high adherence, with a score of 8–12; intermediate adherence, with a score of 4–7; and low adherence, with a score of 0–3 (38).

#### 2.2.3 Anthropometric measurements

Body mass index was used to assess the nutritional status of the adolescents on the basis of their weight and height. Body mass index is calculated by dividing a person’s weight in kilograms by the square of their height in meters (kg/m²).

#### 2.2.4 Justification for including covariates

The relationships of SN use and SN addiction with self-esteem in adolescents could be influenced by a variety of factors. When assessing this relationship, it is crucial to account for several variables that may mediate or moderate the effects of SN use. The covariates included in this study—such as sex, age, socioeconomic status, sleep duration, physical activity, sedentary behavior, body mass index, and adherence to the Mediterranean diet—are well-established factors that could impact the psychological and physical well-being of adolescents (12–15).

#### 2.2.5 Statistical analysis

Both statistical (Shapiro-Wilk test) and visual (quantile-quantile and density plots) normality tests were performed to determine the normal distribution of the variables. The results confirmed that the variables had a normal distribution, thus justifying the use of parametric statistical techniques in the analysis. Means (M) and standard deviations (SD) are reported for quantitative variables, and frequencies (n) and percentages (%) are reported for qualitative variables. Since preliminary analyses revealed no interaction when SN use or SN addiction and sex were tested in relation to self-esteem, no sex-divided analysis was performed. Generalized linear models (GLMs) with Gaussian distributions were performed to calculate unstandardized beta coefficients (*B*) and their 95% confidence intervals (95% CI). The *post hoc* power analysis was conducted for a multiple linear regression with 10 predictors, an expected *R*² of 0.30, and a sample size of 632 participants. The analysis indicated that the sample size required for detecting a moderate effect at a power of 0.80 and α = 0.05 was 48 participants. Therefore, the included sample size of 632 would be sufficiently powered to detect effects of this size with high confidence. Age, sex, socioeconomic status, sleep duration, physical activity, sedentary behavior, adherence to the Mediterranean diet, and BMI were entered as covariates. All the statistical analyses were conducted via R software (Version 4.3.2) (R Core Team, Vienna, Austria) and RStudio (2023.09.1 + 494) (Posit, Boston, MA, USA). A p value less than 0.05 was regarded as statistically significant.

## 3 Results

**Table 1** shows the descriptive data of the 632 study participants. Instagram was the SN most widely used by adolescents, whereas Facebook was the least used SN. In terms of SN addictive behaviors, tolerance (63.3%) was the predominant behavior presented by the participants, followed by mood modification (45.9%), relapse (34.8%), salience (24.1%), withdrawal (23.9%) and, finally, conflict (22.9%).

**Table 1:** Main characteristics of the study participants (n=632).

Age (years)	14.0 (1.5)
Sex	
Boys (%)	272 (43.0)
Girls (%)	360 (57.0)
FAS-III (score)	8.1 (2.1)
Overall sleep duration (minutes)	493.2 (54.8)
YAP-S physical activity (score)	2.6 (0.7)
YAP-S sedentary behaviors score)	2.6 (0.6)
KIDMED (score)	6.6 (2.5)
Body mass index (kg/m^2^)	22.7 (4.7)
SN use	
Facebook use (score)	1.2 (0.7)
Twitter use (score)	1.5 (0.9)
Instagram use (score)	3.5 (1.3)
Snapchat use (score)	1.4 (0.9)
TikTok use (score)	3.3 (1.5)
WhatsApp use (score)	3.5 (1.1)
SN use (score) ^a^	13.3 (3.6)
SN addictive behaviors	
Tolerance (yes, %)	400 (63.3)
Salience (yes, %)	152 (24.1)
Mood modification (yes, %)	290 (45.9)
Relapse (yes, %)	220 (34.8)
Withdrawal (yes, %)	151 (23.9)
Conflict (yes, %)	145 (22.9)
SNAddS-6S (score) ^b^	3.3 (1.5)
Rosenberg Self-Esteem Scale (score)	26.3 (4.3)

FAS-III, Family Affluence Scale-III; KIDMED, Mediterranean Diet Quality Index in Children and Adolescents; SN, Social Networks; SNAddS-6S, Social Network Addiction Scale-6 Symptoms; YAP-S, Spanish version of the Youth Activity Profile. The values are means (standard deviations) unless otherwise indicated.

a SN use score including Facebook, Twitter, Instagram, Snapchat, and TikTok.

b Calculated as the mean number of addictive behaviors toward the SN shown.

**Figure 1:**
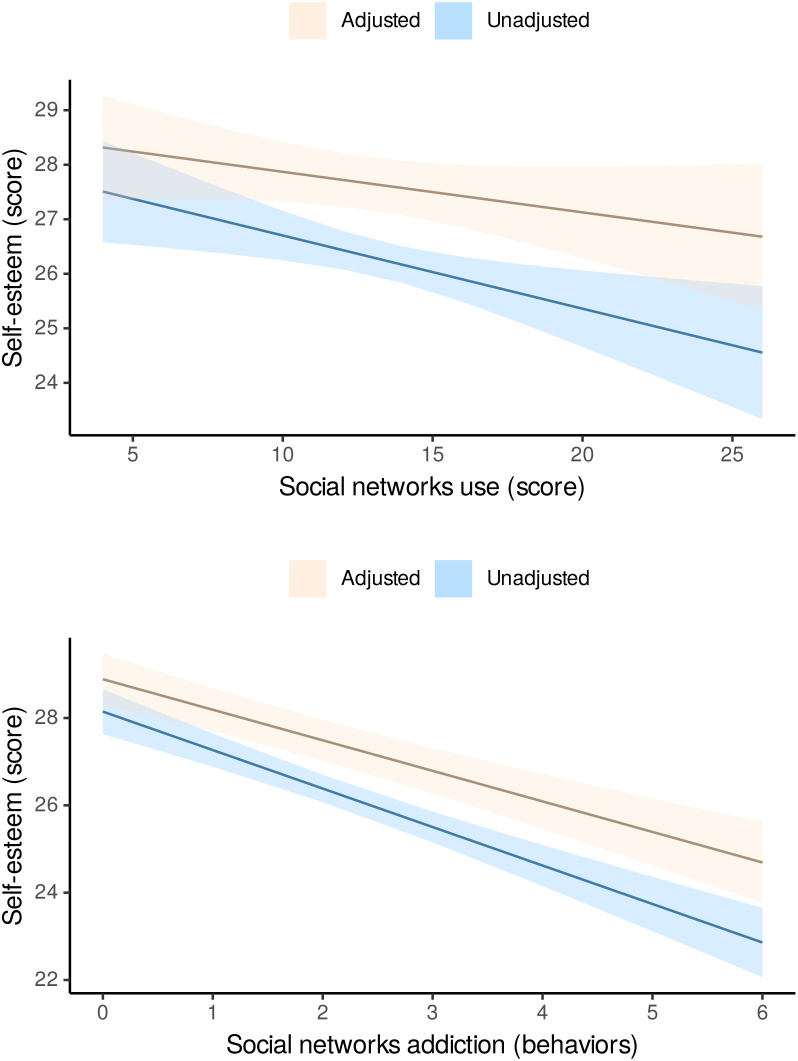
Estimated marginal means of self-esteem based on social network use or addictive behaviors toward their use in Spanish adolescents.

**Table 2** shows the GLMs examining the relationships of SN use or addiction to SN with self-esteem in adolescents. Furthermore, **Figure 1** shows the estimated marginal means of self-esteem based on the SN use score and the number of SN addictive behaviors, as derived from the GLMs. In the adjusted model where the different covariates (age, sex, socioeconomic level, sleep duration, physical activity, sedentary behavior, adherence to the Mediterranean diet and BMI) were included, the association between SN use and self-esteem was no longer significant (*B =* -0.064; 95% CI -0.157 to 0.029; *p = 0*.178). For SN addiction, an increase in each addictive behavior was associated with a significant decrease in self-esteem (*B =* -0.699; 95% CI -0.890 to -0.508; *p <* 0.001).

**Table 2:** Generalized linear models for the relationships of social network use or addictive behavior with social network use and self-esteem in adolescents.

Predictors	Self-esteem (score)	
	Model 0	Model 1
	*B* (95% CI), *p* value	*B* (95% CI), *p* value
SN use (for each point)	-0.134 (-0.227 to -0.041), *p* = 0.005	-0.064 (-0.157 to 0.029), *p* = 0.178
SN addictive behaviors (for each behavior)	-0.881 (-1.070 to -0.692), *p* < 0.001	-0.699 (-0.890 to -0.508), *p* < 0.001

The data are expressed as unstandardized beta (*B*) coefficients, 95% confidence intervals (CIs), and *p* values. Model 0: unadjusted. Model 1: adjusted for age, sex, socioeconomic level, sleep duration, physical activity, sedentary behavior, adherence to the Mediterranean diet, and body mass index.

**Table 3** shows the associations between the use of the different SNs examined and adolescent’s self-esteem. A significant negative association was found between Twitter use and self-esteem (*B* = -0.356; 95% CI -0.695 to -0.017; *p* = 0.040), suggesting that greater Twitter use is linked to lower self-esteem. No significant associations were detected for Facebook use (*B* = -0.213; 95% CI -0.672 to 0.246; *p* = 0.363), Instagram use (*B* = -0.159; 95% CI -0.410 to 0.092; *p* = 0.217), Snapchat use (*B* = -0.195; 95% CI -0.556 to 0.166; *p* = 0.288), or TikTok use (*B* = -0.170; 95% CI -0.390 to 0.050; *p* = 0.128). Although WhatsApp use showed a positive trend toward higher self-esteem, this association was not statistically significant (*B* = 0.279; 95% CI -0.013 to 0.571; *p* = 0.062). With respect to addictive behaviors toward SNs, mood modification showed the strongest negative association with self-esteem (B = -2.580; 95% CI -3.192 to -1.968; *p* < 0.001), followed by conflict (B = -1.410; 95% CI -2.147 to -0.673; *p* < 0.001), relapse (B = -1.350; 95% CI -1.999 to -0.701; *p* < 0.001), tolerance (B = -0.928; 95% CI -1.596 to -0.260; *p* = 0.007), salience (B = -0.892; 95% CI -1.623 to -0.161; *p* = 0.017), and finally, withdrawal behaviors (B = -0.170; 95% CI -1.905 to -0.435; *p* = 0.002), all of which were significantly negatively associated with self-esteem in adolescents.

**Table 3:** Generalized linear models for the associations of each individual social network used or WhatsApp and each addictive behavior with social network use and self-esteem in adolescents.

Predictors	Self-esteem (score)
SN used	
Facebook use (per point)	*B* = -0.213; 95% CI -0.672 to 0.246; *p* = 0.363
Twitter use (per point)	*B* = -0.356; 95% CI -0.695 to -0.017, *p* = 0.040
Instagram use (per point)	*B* = -0.159; 95% CI -0.410 to 0.092, *p* = 0.217
Snapchat use (per point)	*B* = -0.195; 95% CI -0.556 to 0.166, *p* = 0.288
TikTok use (per point)	*B* = -0.170; 95% CI -0.390 to 0.050, *p* = 0.128
WhatsApp use (per point)	*B* = 0.279; 95% CI -0.013 to 0.571, *p* = 0.062
SN addictive behavior	
Tolerance (yes)	*B* = -0.928; 95 CI % -1.596 to -0.260, *p* = 0.007
Salience (yes)	*B* = -0.892; 95 CI % -1.623 to -0.161, *p* = 0.017
Mood modification (yes)	*B* = -2.580; 95 CI % -3.192 to -1.968, *p* < 0.001
Relapse (yes)	*B* = -1.350; 95 CI % -1.999 to -0.701, *p* < 0.001
Withdrawal (yes)	*B* = -0.170; 95 CI % -1.905 to -0.435, *p* = 0.002
Conflict (yes)	*B* = -1.410; 95 CI % -2.147 to -0.673, *p* < 0.001

The data are expressed as unstandardized beta values (*B*), 95% confidence intervals (CIs), and *p* values. Age, sex, socioeconomic level, sleep duration, physical activity, sedentary behavior, adherence to the Mediterranean diet, and body mass index were adjusted for. SN, social network.

## 4 Discussion

The results obtained in this study highlight the significant relationships of the use of SN (i.e., Twitter) with self-esteem in adolescents. Furthermore, certain addictive behaviors related to SN use, such as tolerance, salience, mood modification, relapse, withdrawal, and conflict, were particularly linked to lower self-esteem. In contrast to our hypothesis, our findings suggest that while general SN use was not directly associated with lower self-esteem after adjusting for key covariates, some addictive behaviors related to SN use were strongly linked to decreased self-esteem. This distinction underscores the importance of differentiating between casual and problematic SN use when evaluating its psychological effects. The scientific literature regarding the relationships of SN use and SN addiction with self-esteem is not conclusive. For example, a review by Lin (39) found a significant correlation between online SN use and various aspects of adolescent mental health, including mood, life satisfaction, overall well-being, and self-esteem. Similarly, another study by Hawi et al. (40) indicated that SN addiction had an inverse association with self-esteem among university students. Conversely, a previous systematic review reported that online SNs are positively associated with mood, life satisfaction, and loneliness in adolescents but not with self-esteem (11). Furthermore, Glušac et al. (41) reported that SN use and self-esteem are not related but that a more positive attitude toward education, standards, and people affects self-esteem. Additionally, a previous study by Valkenburg et al. (42) noted that the frequency of use of a friend networking site has an indirect effect on adolescent’s social self-esteem and well-being, with positive feedback enhancing self-esteem and well-being and negative feedback decreasing it. Moreover, Villegas Domínguez et al. (43) noted that Instagram was associated with low self-esteem, whereas SN addiction, in general, was not associated with low self-esteem in their study among university students.

There are several potential explanations for the divergent results found regarding the associations between SN use and SN addiction with self-esteem. First, it is important to distinguish between “use” and “addiction” to SNs (17). “Use” refers to regular, balanced interaction with SNs that do not interfere with daily life, such as academic or social responsibilities, and is not compulsive. In contrast, “addiction” involves compulsive use that disrupts daily life, characterized by constant checking, prolonged online engagement, and anxiety when access is restricted, which often leads to negative effects in different areas of life. Therefore, it is possible that the use of SNs itself is not harmful until it reaches a certain level that results in consequences (such as symptoms and/or behaviors of addiction), becoming deleterious for mental health and impacting self-esteem (11). Second, these variations could be influenced by additional factors, such as the associations among different variables (12–15). It has been previously suggested that other contextual or personal variables may play a critical role in moderating the effects of SN use on mental health outcomes in adolescents (44). In fact, our results regarding the relationship between SN use and adolescent self-esteem could support, at least in part, this notion since the association became nonsignificant after adjusting for several covariates. Hence, while further research is needed to clarify the underlying mechanisms and the nature of these relationships, several factors may help explain the results.

The association between SN addiction and lower self-esteem may be explained by multiple psychological mechanisms. One key factor is social comparison, as SN platforms expose adolescents to highly curated and idealized portrayals of others (53, 54). This exposure can lead to negative self-evaluations (45), where adolescents perceive themselves as inferior in aspects such as physical appearance, social status, or personal achievements, ultimately lowering their self-esteem (56). In particular, appearance-focused SN use has been linked to lower body satisfaction and well-being among adolescents, with similar effects observed across sexes (57). Additionally, digital validation plays a crucial role. Adolescents increasingly rely on comments, follower counts, and “likes” as indicators of social acceptance and self-worth (59, 60). The pursuit of external validation, particularly in appearance-related contexts, has been associated with negative outcomes such as disordered eating, substance use, aggression, and stress (61). When adolescents fail to achieve the anticipated level of engagement, they may experience feelings of rejection or inadequacy (60), contributing to a decline in self-esteem. Over time, this reliance on SN feedback can create a cycle where self-worth becomes contingent on digital interactions (62). Finally, individual differences likely moderate these associations (46). For example, adolescents with higher self-esteem or greater emotional resilience may be less affected by negative social comparisons (47), whereas those with preexisting body image concerns or greater social anxiety might be more vulnerable (48). Similarly, personality traits such as neuroticism and extraversion could influence the extent to which digital validation impacts self-worth (49).

Additionally, prolonged SN use may reduce face-to-face interactions (50), which are essential for developing social skills and emotional support. This reduction can negatively impact adolescent’s ability to engage in meaningful social exchanges, particularly affecting their emotional, instrumental, and communicative skills, whereas cognitive social skills appear to be less affected (51). Despite having numerous online connections, adolescents may still experience social isolation due to the lack of meaningful real-world relationships (52). This isolation has been linked to an increased risk of depressive symptoms, suicide attempts, and low self-esteem (53). Therefore, the combination of reduced face-to-face interactions and limited authentic social exchanges may contribute to feelings of loneliness and diminished self-esteem.

Regarding the specific SN, we found that the use of Twitter, but not other studied SNs, was associated with lower self-esteem. These results could suggest that the impact of SN on self-esteem in adolescents can differ depending on the specific SN utilized (54). This finding could be attributed to several factors, including the different characteristics of every SN and the nature of the interactions promoted by them (e.g., positive versus negative feedback) (55, 56). In this sense, first, Twitter’s fast-paced and often contentious environment can expose users to negative comments and criticism more readily than other platforms, which could harm their self-esteem (57). Second, the constant social comparison on Twitter, where users frequently measure their opinions, achievements, and popularity against others, can lead to feelings of inferiority (58). Third, the overwhelming amount of information and opinions on Twitter can be stressful and contribute to lower self-esteem (59). Fourth, the superficial nature of interactions on Twitter, which are typically brief and less meaningful, may also limit the social and emotional support that users receive (60). Finally, exposure to sensitive or negative content on Twitter could negatively impact user’s emotional well-being and self-esteem (61).

Given that social comparison, digital validation, and reduced face-to-face interactions are key psychological mechanisms underlying the negative impact of SN addiction on self-esteem, intervention strategies should directly target these risk factors to promote healthier digital habits among adolescents. Policymakers, educators, and parents play a key role in fostering healthier SN use and mitigating its negative effects. Educational institutions should implement digital literacy programs that teach adolescents how to navigate SN critically, recognizing unrealistic portrayals and minimizing harmful social comparisons (54). These programs should also promote self-regulation strategies to prevent excessive use and dependence on online validation (4). Research has shown that media literacy interventions can improve resilience against the negative psychological impacts of SN use, making this an essential component of educational efforts (9). Parents should be encouraged to foster open communication about SN experiences, helping adolescents differentiate between online perceptions and reality (5). Setting boundaries, such as screen time limits and technology-free zones (e.g., during meals or before bedtime), may also reduce excessive dependence on SN and encourage healthier social interactions (26, 62). Studies indicate that structured parental guidance on SN use could positively impact adolescent self-esteem and well-being (6, 13). Counseling services in schools and community centers should integrate discussions on digital well-being, self-esteem, and online behavior (10). Encouraging adolescents to follow positive and educational content, rather than appearance-focused or highly curated social media, could help mitigate the negative effects of excessive SN use (19, 40). Research suggests that excessive focus on appearance-related content on SNs is associated with body dissatisfaction and lower self-esteem in adolescents (8, 25). Policy efforts should also consider evidence from existing digital well-being initiatives, such as educational programs in European countries (63) and screen time restrictions implemented in China (64), which could have certain effectiveness in reducing problematic SN use and its psychological consequences. Policymakers could also support awareness campaigns about the risks of SN addiction and its impact on self-esteem (7). By integrating these strategies, it may be possible to create a healthier digital environment where adolescents can engage with SNs in a balanced way, minimizing risks to their self-esteem and mental well-being. Future research should explore the effectiveness of such interventions and policy measures in promoting digital well-being among adolescents.

This study has several limitations. First, the cross-sectional design precludes the establishment of causality between SN use and self-esteem, as it provides only a snapshot of the relationship at one point in time. Second, the reliance on self-reported data introduces the possibility of social desirability bias or inaccuracies in responses, particularly in sensitive topics such as the frequency of SN use, self-esteem and SN addiction. Third, the study does not account for potential cultural or socioeconomic differences, which could play a significant role in the relationship between SN use and self-esteem. Future studies with longitudinal designs and a more comprehensive set of control variables are needed to better understand the causal pathways and mitigate these limitations. However, this study has several strengths. The study examined overall SN use as well as specific platforms and addictive behaviors, offering insights into how these platforms and behaviors are linked to self-esteem. In addition, the use of generalized linear regression analyses, adjusted for various sociodemographic, anthropometric and lifestyle factors, strengthens the study’s conclusions by controlling for potential confounding variables.

## 5 Conclusions

SN use and SN addictive behaviors could significantly affect adolescent self-esteem. The findings suggest that the use of Twitter and certain addictive behaviors, such as tolerance, salience, mood modification, relapse, withdrawal, or conflict are particularly linked to lower self-esteem. These results highlight the need to develop strategies aimed at promoting healthy engagement with SN platforms and fostering adolescent’s psychological well-being.

## Data Availability

The raw data supporting the conclusions of this article will be made available by the authors, without undue reservation.
